# *In Vivo* three-dimensional kinematics of normal knees during sitting sideways on the floor

**DOI:** 10.1186/s12891-022-05267-z

**Published:** 2022-04-06

**Authors:** Kenichi Kono, Takaharu Yamazaki, Shoji Konda, Hiroshi Inui, Sakae Tanaka, Kazuomi Sugamoto, Tetsuya Tomita

**Affiliations:** 1 Department of Orthopaedic Surgery, Faculty of Medicine, The University of Tokyo, 7-3-1 Hongo, Bunkyo-ku, Tokyo, 113-0033 Japan; 2 Department of Orthopaedic Biomaterial Science, Osaka University Graduate School of Medicine, 2-2 Yamada-oka, Suita, Osaka 565-0871 Japan; 3 Information Technology Course, Faculty of Engineering, Saitama Institute of Technology, 1690 Fusaiji, Fukaya, Saitama 369-0293 Japan; 4 Department of Health and Sport Sciences, Osaka University Graduate School of Medicine, 2-2 Yamada-oka, Suita, Osaka 565-0871 Japan

**Keywords:** Sitting sideways, Kneeling, Kinematics, Normal knee

## Abstract

**Background:**

The normal knee kinematics during asymmetrical kneeling such as the sitting sideways remains unknown. This study aimed to clarify *in vivo* kinematics during sitting sideways of normal knees.

**Methods:**

Twelve knees from six volunteers were examined. Under fluoroscopy, each volunteer performed a sitting sideways. A two-dimensional/three-dimensional registration technique was used. The rotation angle, varus-valgus angle, anteroposterior translation of the medial and lateral sides of the femur relative to the tibia, and kinematic pathway in each flexion angle was evaluated.

**Results:**

Bilateral knees during sitting sideways showed a femoral external rotation relative to the tibia with flexion (ipsilateral: 13.7 ± 3.5°, contralateral: 5.8 ± 6.8°). Whereas the ipsilateral knees showed valgus movement of 4.6 ± 2.5° from 130° to 150° of flexion, and the contralateral knees showed varus movement of -3.1 ± 4.4° from 110° to 150° of flexion. The medial side of the contralateral knees was more posteriorly located than that of the ipsilateral knees beyond 110° of flexion. The lateral side of the contralateral knees was more anteriorly located than that of the ipsilateral knees from 120° to 150° of flexion. In the ipsilateral knees, a medial pivot pattern followed by a bicondylar rollback was observed. In the contralateral knees, no significant movement followed by a bicondylar rollback was observed.

**Conclusion:**

Even though the asymmetrical kneeling such as sitting sideways, the knees did not display asymmetrical movement.

## Background

Many studies reported the kinematics of normal knees is activity-dependent [1–7]. Therefore, it is important to evaluate each activity.

Kneeling is one of the most common activities of daily living such as sitting on the floor, gardening, and praying. Concerning the kinematics, several studies demonstrated that a femoral external rotation with a medial pivot is observed with the knee flexion during kneeling [3, 4]. There are many kinds of sitting styles such as sitting cross-legged, seiza-sitting, and sitting sideways. In addition, the kinematics during sitting cross-legged is different from that of kneeling and squatting [4]. However, most of the sitting styles evaluated previously were symmetrical [2–4, 8]. Therefore, the kinematics during asymmetrical sitting such as the sitting sideways remains unknown.

Especially in Asian and Middle-Eastern countries, people habitually performed the sitting with deep-knee-bend. Therefore, the patients after total knee arthroplasty (TKA) also desire sitting with deep-knee-bend [9]. Whereas, many TKA implants did not create the normal knee kinematics [10]. Also, several studies demonstrated that the achievement of normal-like kinematics following TKA is related to the high clinical outcome scores [11–14]. Thus, the evaluation of the normal knee kinematics of side sitting is meaningful to improve the clinical outcome.

Moreover, several studies reported that kneeling is a risk factor of knee osteoarthritis (OA) [15, 16]. The evaluation of asymmetrical kneeling is also important to elucidate the mechanism of OA.

This study aimed to clarify *in vivo* kinematics during sitting sideways of normal knees. The hypothesis of this study was that *in vivo* kinematics during sitting sideways was different between the ipsilateral knees and contralateral knees, in other words, the ipsilateral knees showed femoral external rotated, valgus, and medial pivot motion with flexion, on the other hand, the contralateral knees showed femoral internal rotated, varus, and lateral pivot motion with flexion.

## Methods

Twelve knees from six volunteers were examined. All of the volunteers were Japanese males and provided written informed consent to participate in this study. All participants gave written informed consent to the experimental procedure, which was approved by the ethics committee of Osaka University (Number 13106) in Osaka, Japan and in accordance with the Declaration of Helsinki. Inclusion criteria were non-osteoarthritic knees on computed tomography (CT) and no symptomatic knees. Exclusion criteria were post-injury knees (fracture, ligament injury, and meniscus injury) and inflammatory arthritic knees. At the time of examination, the mean age was 37.3 ± 7.6 years. The mean body height was 169.9 ± 5.2 cm. The mean body weight was 64.2 ± 5.2 kg. All of the values were expressed as mean ± standard deviation (SD).

**Fig. 1:**
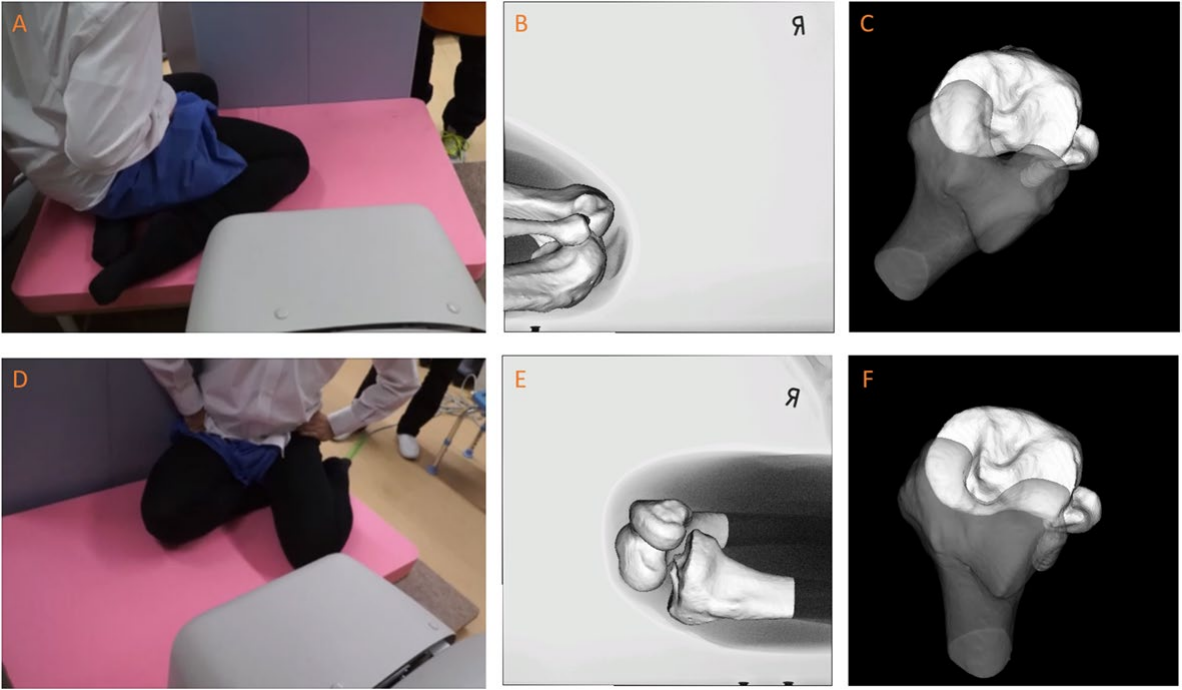
Sitting sideways under fluoroscopy. **A** Evaluation of the ipsilateral knee. **B** Fluoroscopic image after 2D/3D registration (ipsilateral knee). **C** Axial view after 2D/3D registration (ipsilateral knee). **D** Evaluation of the contralateral knee. **E** Fluoroscopic image after 2D/3D registration (contralateral knee). **F** Axial view after 2D/3D registration (contralateral knee)

Under fluoroscopy, each volunteer performed a sitting sideways from kneeling position to maximal high flex position at a natural pace (Fig. 1). The volunteers were instructed only to flex their knees as much as possible. The subjects were not instructed regarding the leg position because it was found that this resulted in an unnatural motion. They practiced the motion several times before recording. The right and left knee motions were separately recorded. In other words, a total of 4 motions (the right leg on the inside and outside, and the left leg on the inside and outside) were recorded in each volunteer. The sequential motion was recorded as a series of digital X-ray images (1024 × 1024 × 12 bits/pixel, 7.5-Hz serial spot images as a DICOM file) using a 17-inch (43-cm) flat panel detector system. Furthermore, all images were processed by dynamic range compression, thereby enabling edge-enhanced images. To estimate the spatial position and orientation of the knee, a two-dimensional/three-dimensional (2D/3D) registration technique [4, 17] was employed.

Three-D bone models were created from CT and used for the computer-aided design (CAD) models. The CT was taken from each subject. Estimation accuracy for the relative motion between 3D bone models was ≤ 1° in rotation and ≤ 1 mm in translation [4].

A local coordinate system at the bone model was produced according to a previous study [18]. Knee rotations were described using the joint rotational convention of Grood and Suntay [19]. Femoral rotation angle relative to the tibia, varus-valgus angle, and anteroposterior (AP) translation of the medial sulcus (medial side) and lateral epicondyle (lateral side) of the femur on the plane that is perpendicular to the tibial mechanical axis in each flexion angle were evaluated [4]. AP translation was calculated as a percentage relative to the proximal AP dimension of the tibia [4]. External rotation was denoted as positive and internal rotation as negative. Valgus was denoted as positive and varus as negative. Positive and negative values of AP translation were described as anterior and posterior to the axis of the tibia, respectively.

### Statistical analyses

Results were analysed using SPSS version 24 (IBM Corp., Armonk, NY, USA). Repeated measure analysis of variance (ANOVA) and post hoc pairwise comparison (Bonferroni test) was used to analyse all evaluation items. A *p*-value < 0.05 was considered statistically significant. Moreover, a power analysis using EZR [20] indicated that 11 knees would be required for an alpha set at 0.05 and power at 0.8.

## Results

### Flexion, rotation, and varus-valgus angle

Ipsilateral knees during sitting sideways were gradually flexed from 98.4 ± 6.8° to 150.8 ± 4.5°, and contralateral knees during sitting sideways were gradually flexed from 101.7 ± 6.2° to 155.2 ± 4.8°.

**Fig. 2:**
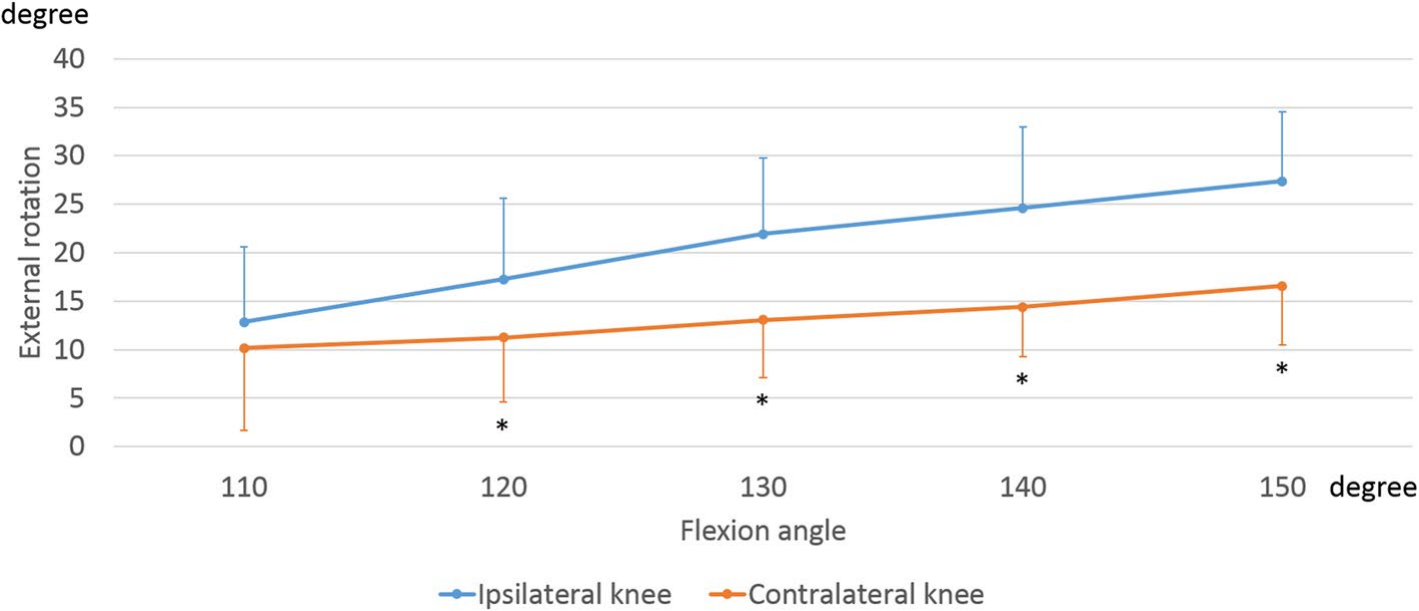
Rotation angle during sitting sideways. The markers indicate the femoral rotation relative to the tibia. *, significant differences between ipsilateral knees and contralateral knees (*p* < 0.05)

In the ipsilateral knees, the femurs displayed an external rotation of 13.7 ± 3.5° relative to the tibia from 110° to 150° of flexion. In the contralateral knees, the femurs displayed an external rotation of 5.8 ± 6.8° relative to the tibia from 110° to 150° of flexion (Fig. 2). From 120° to 150° of flexion, the femoral external rotation of contralateral knees was significantly smaller than that of ipsilateral knees (120°: *p* = 0.008, 130°: *p* = 0.001, 140°: *p* < 0.001, 150°: *p* < 0.001).

**Fig. 3:**
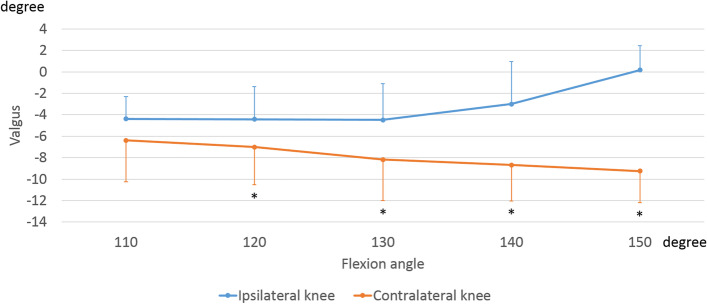
Varus-valgus angle during sitting sideways. The markers indicate the femoral varus-valgus movement relative to the tibia. *, significant differences between ipsilateral knees and contralateral knees (*p* < 0.05)

Regarding the varus-valgus angle, the ipsilateral knees showed the valgus movement of 4.6 ± 2.5° from 130° to 150° of flexion. On the other hand, contralateral knees showed varus movement of -3.1 ± 4.4° from 110° to 150° of flexion (Fig. 3). From 120° to 150° of flexion, the contralateral knees showed significantly varus position than ipsilateral knees (120°: *p* = 0.006, 130°: *p* = 0.005, 140°: *p* = 0.001, 150°: *p* < 0.001).

### AP translation

**Fig. 4:**
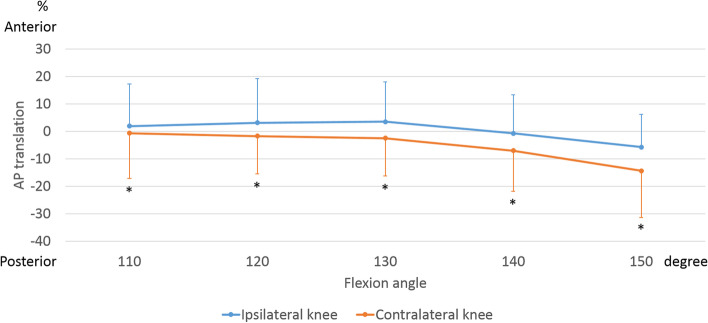
Anteroposterior (AP) translation of the femoral medial sulcus during sitting sideways. AP translation was calculated as a percentage relative to the AP length of the tibia. *, significant differences between ipsilateral knees and contralateral knees (*p* < 0.05)

The AP translation of the medial side of the ipsilateral femur indicated 10.4 ± 7.0% posterior movement from 130° to 150° of flexion. The AP translation of the medial side of the contralateral femur indicated 11.0 ± 8.6% posterior movement from 130° to 150° of flexion (Fig. 4). Beyond 110° of flexion, the medial side of the contralateral knees was significantly more posteriorly located than that of the ipsilateral knees (*p* = 0.011).

**Fig. 5:**
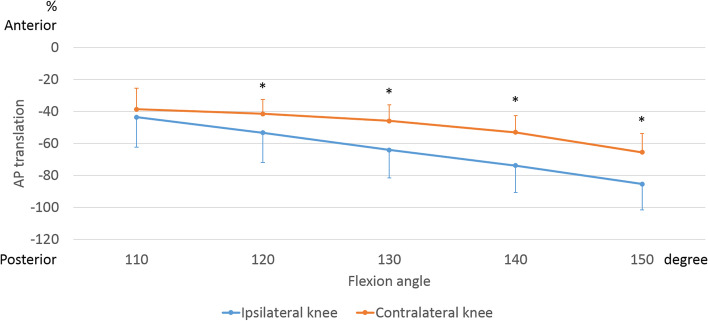
Anteroposterior (AP) translation of the femoral lateral epicondyle during sitting sideways. AP translation was calculated as a percentage relative to the AP length of the tibia. *, significant differences between ipsilateral knees and contralateral knees (*p* < 0.05)

The AP translation of the lateral side of the ipsilateral femur indicated 40.0 ± 6.6% posterior movement beyond with 110° of flexion. The AP translation of the lateral side of the contralateral femur indicated 20.2 ± 4.4% posterior movement from 130° to 150° of flexion (Fig. 5). From 120° to 150° of flexion, the lateral side of the contralateral knees was significantly more anteriorly located than that of the ipsilateral knees (120°: *p* = 0.008, 130°: *p* < 0.001, 140°: *p* < 0.001, 150°: *p* < 0.001).

### Kinematic pathway (Fig. 6)

**Fig. 6:**
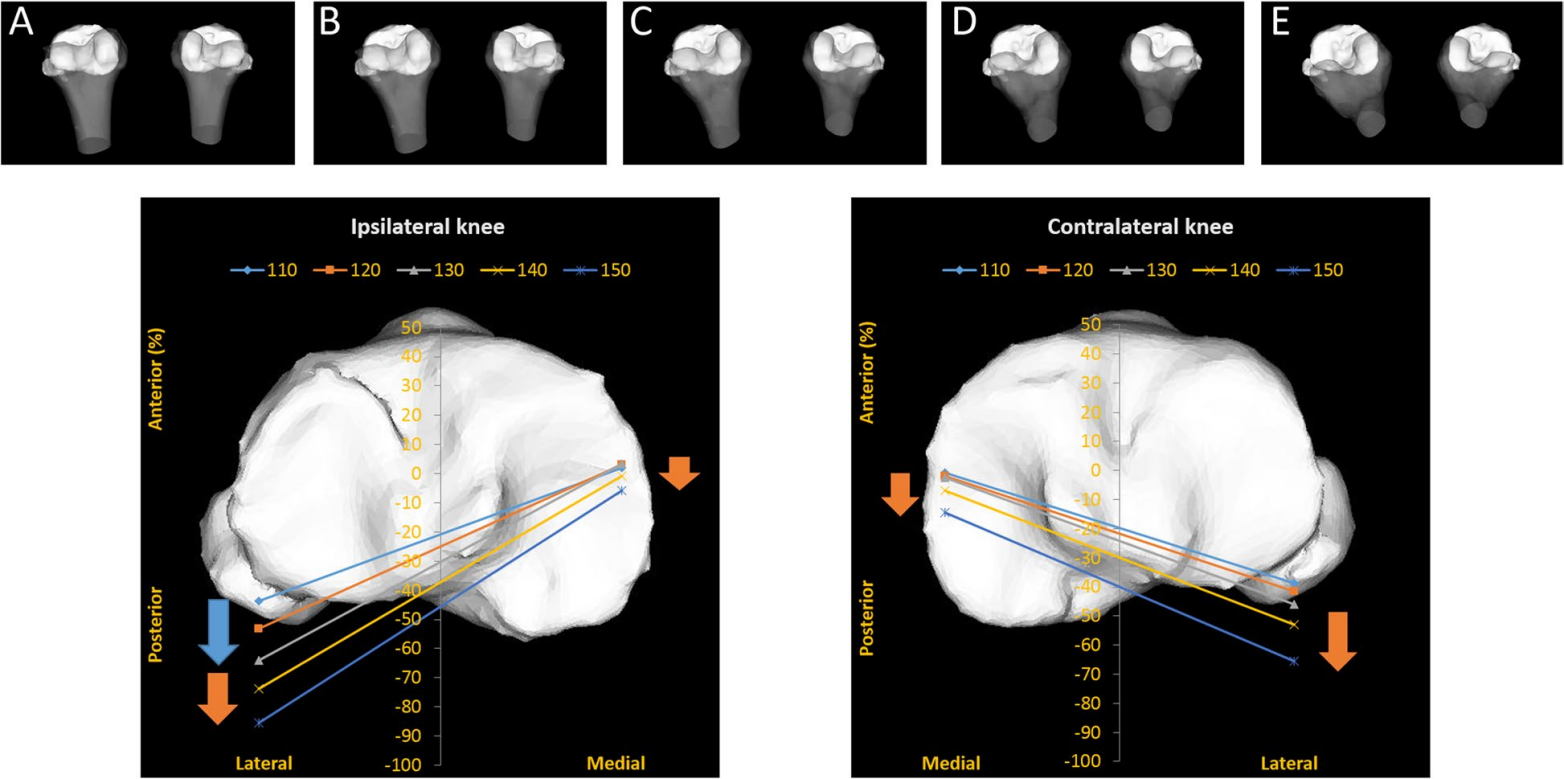
Kinematic pathway of ipsilateral knees and contralateral knees during sitting sideways. Above row indicates it each flexion angle (A: 110°, B: 120°, C: 130°, D: 140°, E: 150°) Bottom row indicates the whole movement from 110° to 150° of flexion. Blue and orange arrows indicate the direction of the movement. Left knee is ipsilateral and right knee is contralateral. Ipsilateral knee Blue arrow: From 110° to 130° of flexion. Orange arrow: From 130° to 150° of flexion. Contralateral knee Orange arrow: From 130° to 150° of flexion

In the ipsilateral knees, the difference between the medial and lateral sides of the femur reflected a medial pivot pattern from 110° to 130° of flexion. From 130° to 150° of flexion, a bicondylar rollback was observed. In the contralateral knees, no significant movement was observed from 110° to 130° of flexion. From 130° to 150° of flexion, a bicondylar rollback was observed.

## Discussion

This is the first study to evaluate the *in vivo* knee kinematics of normal volunteers during asymmetrical kneeling using the CAD model of fluoroscopically captured images. Regarding the varus-valgus angle, the ipsilateral knees during sitting sideways showed valgus movement, on the other hand, the contralateral knees during sitting sideways showed varus movement. This fact was as hypothesized. Whereas, regarding rotation angle and AP translation, the result of this study was contrary to the hypothesis. In other words, the bilateral knees showed the femoral external rotation, additionally, the contralateral knees did not show a lateral pivot motion. These facts suggest that regardless of the varus-valgus movement, normal knees during asymmetrical kneeling display a femoral external rotation. Moreover, a lateral pivot motion was not observed even though the contralateral knees during asymmetrical kneeling. Namely, even though the asymmetrical kneeling, the knees did not display asymmetrical movement. Previous studies reported that the rate of medial OA knees in Asian countries was higher than that of Western countries [21]. One of the reasons is a difference in their habit. In other words, Asian people habitually performed the sitting with deep-knee-bend. The kneeling performance displayed sharp medial pivot motion [4], even though the asymmetrical kneeling. These facts might induce a high rate of medial OA knees in Asian people. However, the extent of rotation angle and AP translation was significantly different between the ipsilateral knees and contralateral knees; the femoral external rotation of contralateral knees was smaller than that of ipsilateral knees, the medial side of contralateral knees was located more posterior than that of ipsilateral knees, and the lateral side of contralateral knees was located more anterior than that of ipsilateral knees. Therefore, although the kinematics of contralateral knees during sitting sideways was not contrasted with that of ipsilateral knees perfectly during sitting sideways, it might be more laterally constrained movement.

Murakami et al. reported that the knees during an asymmetrical activity such as golf swing displayed asymmetrical movement, unlike this study. The golf swing is a closed-kinetic-chain activity, whereas the sitting sideways is an open-kinetic-chain activity. This suggests that even though the asymmetrical activities, the kinematics is different depending on each activity. Moreover, in sitting sideways, the respective movement of femur and tibia may be different between the ipsilateral knees and contralateral knees.

The previous study that evaluated the knee kinematics during symmetrical kneeling has reported that the range of femoral external rotation with flexion was 14.8 ± 3.8°, and the range of lateral AP translation with flexion was 40.2 ± 10.2% [4]. The rotation and lateral AP translation of ipsilateral knees during sitting sideways were similar to that of normal knees during symmetrical kneeling [3, 4]. On the other hand, the rotation and lateral AP translation of contralateral knees during sitting sideways were smaller than those of normal knees during symmetrical kneeling [3, 4].

Regarding the kinematic pathway, a bicondylar rollback was observed from 130° to 150° of flexion in both the ipsilateral and contralateral knees during sitting sideways. The previous studies demonstrated that normal knee kinematics showed a medial pivot pattern during kneeling [3, 4]. These suggest that the normal knee kinematics during kneeling is different between symmetrical activity and asymmetrical activity in high-flexion.

Gladnick et al. reported that although from extension to 90° of flexion normal knee exhibited an increase in varus-valgus laxity as the flexion angle increase, the variability of AP translation in response to the varus and valgus load was small [22]. On the other hand, in the current study, the ipsilateral knees displayed valgus movement, whereas the contralateral knees displayed varus movement. Furthermore, the AP translation was different between the ipsilateral knees and contralateral knees. These suggest that during a high-flexion activity more than 90° of flexion, because the varus-valgus laxity increase additionally, the varus and valgus load may affect the AP translation.

This study has some limitations. First, this study analyzed the knee joint kinematics of only Japanese normal males. The knee kinematics of females, OA patients, or the other races may be different. Also, that kinematics may provide additional understandings. However, a previous study demonstrated that the female knee kinematics did not differ from the male kinematics at high flexion [23]. Therefore, the kinematics during sitting sideways in the current study may apply to both genders. Second, in the current study, the right and left knee motion were separately recorded because it is impossible to record the bilateral knees in the flat panel. Therefore, it is capable not to be reflected in the simultaneous knee motion during sitting sideways. Third, a previous study reported that the range of motion (ROM) of the hip joint was related to that of the knee joint during kneeling [2]. Therefore, the ROM of the hip joint may also affect the knee kinematics during sitting sideways. However, the effect of ROM on the hip joint could not be evaluated because it was impossible to record the hip joint in the flat panel.

## Conclusions

Even though the contralateral knee during sitting sideways, the femoral external rotation relative to the tibia was observed. Moreover, a lateral pivot motion was not observed. In other words, the knees during asymmetrical kneeling did not display asymmetrical movement.

## Data Availability

The datasets analyzed during the current study are available from the corresponding author on reasonable request.

## Abbreviations

TKA: Total knee Arthroplasty
OA: Osteoarthritis
CT: Computed tomography
SD: Standard deviations
2D/3D: 2-Dimensional/3-dimensional
CAD: Computer-aided design
AP: Anteroposterior
ANOVA: Analysis of variance
